# Improved Spoken Language Representation for Intent Understanding in a Task-Oriented Dialogue System

**DOI:** 10.3390/s22041509

**Published:** 2022-02-15

**Authors:** June-Woo Kim, Hyekyung Yoon, Ho-Young Jung

**Affiliations:** Department of Artificial Intelligence, Graduate School, Kyungpook National University, Daegu 41566, Korea; kaen2891@knu.ac.kr (J.-W.K.); yhk04150@knu.ac.kr (H.Y.)

**Keywords:** intent understanding, task-oriented dialogue system, spoken dialogue system, speech recognition, spoken language modeling

## Abstract

Successful applications of deep learning technologies in the natural language processing domain have improved text-based intent classifications. However, in practical spoken dialogue applications, the users’ articulation styles and background noises cause automatic speech recognition (ASR) errors, and these may lead language models to misclassify users’ intents. To overcome the limited performance of the intent classification task in the spoken dialogue system, we propose a novel approach that jointly uses both recognized text obtained by the ASR model and a given labeled text. In the evaluation phase, only the fine-tuned recognized language model (RLM) is used. The experimental results show that the proposed scheme is effective at classifying intents in the spoken dialogue system containing ASR errors.

## 1. Introduction

Spoken language understanding (SLU) focuses on catching semantic meanings from voice signals, such as the user’s orders. In other words, the main goal of the SLU task is to understand the meaning of the entire sentence alongside each word, accurately, from the users’ speech. Intent classification, a part of the SLU work, aims at understanding and classifying users’ intents. As advances in artificial intelligence (AI) technology have been widely adapted to natural language processing (NLP), it has improved the performance of text-based SLU systems.

Although several text-based SLU models have been proposed [1,2,3,4,5,6], their approaches are difficult to realize in practical scenarios, where unexpected text errors obtained by automatic speech recognition (ASR) systems are prevalent. This is because erroneous ASR results make the intent classification model unable to accurately understand the users’ intents and orders. Due to the diverse users’ speech in various speaking styles and additive background noise, the ASR system may not be able to properly understand the users’ intents. For these reasons, utilizing the intent classification model trained only from the text-formatted dataset for the spoken dialogue system is not effective. To be used in practical dialogue-oriented systems, an automatic speech recognition-spoken language understanding (ASR-SLU)-based approach that is able to handle the various conditions will be required.

To address the aforementioned problem, several ASR-SLU-based studies [7,8,9,10,11,12] have strived to understand users’ intents based on jointly training with both speech and corresponding text (labeled text). To improve the users’ intents classification, however, these approaches are still vulnerable to the faults in the recognized text obtained by the ASR models. Consequently, it could be considered that there is a fatal flaw in understanding the users’ intentions in practical dialogue-oriented systems. In addition, many speech recognition errors occur, and these cause the SLU systems to have difficulty finding the users’ intents. These problems eventually lead the SLU systems to misclassify users’ intents and could cause critical errors in practical spoken dialogue systems. In light of this, it appears that improved language models are still required for the accurate intent classification in the final state of the spoken dialogue systems.

In this paper, we focus on exploring and analyzing why the recent SLU models in spoken dialogue situations are still weak at understanding utterances from various users and in various conditions. To this end, for each utterance in two SLU datasets [7,13], we obtain the recognized text utilizing a Wav2vec 2.0 ASR model [14], which is the current state-of-the-art. We then report correlations between both character error rate (CER) and word error rate (WER), and intent classification accuracy, where the higher CER and WER lead the intent classification models to yield low performances. In addition, we show the failure case where the language model that is only trained on labeled text performs poorly on the recognized text that contains speech recognition errors.

To cope with the difficulty of intent classification in the practical spoken dialogue systems, we propose a novel and simple method that jointly fine-tunes two pre-trained language models using both labeled text and recognized text. The recognized text includes recognition errors obtained from the user’s speech using ASR systems, and the labeled text means an original script corresponding to the speech. When the labeled text and the recognized text from the users are provided in the training phase, a labeled language model (LLM ) and a recognized language model (RLM) of the proposed model are trained to classify users’ intents gradually and jointly. In contrast, we only use the RLM (spoken language model) in the evaluation phase. Methods introduced in previous studies, such as BERT [15], ALBERT [16], XLNet [17], ELECTRA [18], RoBERTa [19], etc., can be adapted as the two language models of the proposed method.

**Figure 1:**
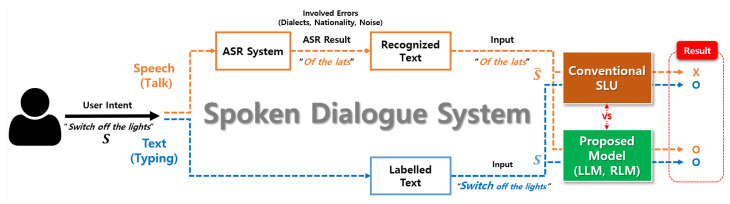
The overall flow of the spoken dialogue system in practical scenarios. Our proposed method could optimally map both recognized text and labeled text to users’ intents.

Figure 1 shows the performance limitations of a spoken dialogue system with ASR errors and presents the process by which our method solves the problem. Our approach could optimally map both speech and the corresponding text to users’ intents by using the pre-trained language models, which have powerful representations in the downstream task. Furthermore, our proposed method could find the users’ intents rapidly and accurately by leveraging pre-trained language models trained on large-scale datasets while previous ASR-SLU-based studies required large computational cost and complex methods.

We show that the proposed scheme outperforms the conventional approach, which is trained on labeled text, and would have achieved low performance on the recognized text dataset with ASR errors. In other words, the results of our experiments explicitly show that the proposed method is flexible in the dialogue-oriented ASR-SLU system. In addition, users are able to transfer their intents to the spoken dialogue system by using only speech or text alone if necessary. As a result, our method is able to contribute to the development of the future spoken dialogue-oriented system for smart home apparatus and portable devices that all humans can enjoy.

The subsequent sections of this paper are organized as follows: In Section 2, we describe the studies related to the SLU and intent classification that are composed of both text-based and speech-text-based approaches. The observations of the issues we raised are illustrated in Section 3 and the proposed method is demonstrated in Section 4. The experimental results and analysis are presented in Section 5. Finally, the conclusion of this paper is drawn in Section 6.

## 2. Related Work

Conventionally, the intent classification task was conducted with models that are solely based on the text datasets. In this section, the traditional methods on intent classification are introduced, and approaches using speech data itself are presented to improve the intent classification of spoken dialogue systems.

### 2.1. Text-Based Intent Classification

With the introduction of the attention mechanism [20], various NLP applications, including the SLU, have used this computation technique. In Liu et al. [1], the advantages of recurrent neural network (RNN) and the attention mechanism were fully realized. As a follow-up study, Goo et al. implemented dual attentions for both slot filling and intent classification tasks with a BiLSTM mechanism for both left and right directions [2]. Unlike previous research that relied heavily on RNN architectures, capsule neural networks have focused on hierarchical relationships between words of token level, allowing the network to naturally catch the most important words conveying the intent of the input sequences [3]. Slot filling and intent detection network [6] applied the statistical conditional random field (CRF) [21] method. The last output of this model is used to finally estimate the CRF for decoding the slots that have the highest probabilities.

On the other hand, with the advancement of parallel computation models based on transformer [22], there have been significant increases in the NLP tasks performances including the SLU field. Owing to the self-attention mechanism with multi-head transformers, NLP problems, such as translation, question and answering, and intent classification, were solved quickly. Bidirectional encoder representations from transformers (BERT) [15] is one of the most successful techniques studied among the language models. BERT is a large-scale data-based language model that learns powerful representation by contaminating the input token with a masked language modeling (MLM) approach and training to reconstruct the corresponding parts. Stack-propagation [4] has not been fully implemented in the whole body of the BERT, but it has shown how effective bidirectional encoders and hierarchical propagation can be. Due to the stacking of the encoder, and both intent classification and slot filling decoders, the stack-propagation model naturally learned semantic knowledge of the input sequence. Chen et al. have shown the powerful performance of its BERT-based model in the intent classification task [5]. Unlike previous studies, which have stacked modules, this method first calculated the slot filling, then utilized its hidden states to classify the intents. The improvement of performance is attributed to BERT, which is pre-trained with an enormous vocabulary.

So far, the aforementioned previous studies have worked to improve the performance of the intent classification from text alone. Since they are not trained with the real human voice, low adaptability in the spoken dialogue systems is expected. Therefore, an approach that can classify the intent using both speech and text is required.

### 2.2. ASR-SLU-Based Intent Classification

Several studies were recently introduced, to solve the limitations of the text-based SLU model for the spoken dialogue system. Chen et al. suggested an end-to-end SLU approach to extract representations from given speech without the ASR system [23]. This architecture is composed of CNN-RNN based networks to allow the model to only use speech signals to find users’ intents directly. Haghani et al. have successfully shown predicting the domains, intents, and orders from audio [24]. Lugosch et al., for an ASR-SLU-based approach, introduced a method to decrease the data requirement of the pre-trained ASR model [7]. Further, they introduced the ASR-SLU dataset, called the fluent speech commands (FSC)-dataset, which was used in our experiments.

As unsupervised learning approaches to extract powerful representations with large-scale datasets have become widely used, many studies have been utilized for the SLU task. Wang et al. used an unsupervised pre-training method with the masking policy for each audio frame to learn acoustic representations [8]. They then fine-tuned the pre-trained model as a downstream task for SLU. Huang et al. proposed a confusion-aware fine-tuning method, which had a similar motivation to ours [9]. Their scheme reduced the similar semantics for phonology of hardly distinguishable words with fine-tuning. Cao et al. showed the pre-trained method for conversational language modeling that enabled the SLU networks to catch the linguistic representations in dialogue styles with the ASR errors [10]. Chung et al. utilized a masking policy approach for the SLU task to jointly pre-train the unpaired speech and text via aligning representations [25]. SpeechBERT was trained via a semi-supervised method, not only representation learning, but also for the intent classification and slot-filling [13]. This model is tested for its demonstration of robustness against ASR errors and extraction of semantic meaning in the input sequence. Qian et al. proposed to integrate an end-to-end ASR encoder and a pre-trained language model encoder into a transformer decoder for the SLU task [26].

As the earlier studies show, ASR-SLU-based methods are widely explored. However, conventional methods not only require pre-training with large-scale datasets of the spoken language model, but they also are incredibly time-consuming to fine-tune. Meanwhile, the proposed method in this paper is very simple and could solve the aforementioned problems without complex computation.

## 3. Observations and Analyses of Intent Understanding Performance Regarding ASR Errors

In this section, we analyze the errors of utterances based on recognized text obtained from the recent state-of-the-art ASR model, Wav2vec 2.0 [14]. To this end, we use the FSC [7] dataset and the Audio-Snips [13] (the authors denoted it as “SNIPS-Multi”, but it will be referred to in this paper as “Audio-Snips”) dataset, which is a widely used SLU benchmark dataset. In the models, which were trained using labeled text and evaluated with labeled and recognized text, we show the issues of the conventional spoken dialogue system that motivate this work through the correlation between the ASR errors and the intent classification performance.

### 3.1. Dataset

The FSC dataset, which is mainly used for the ASR-SLU tasks, contains utterances spoken by both native English speakers and non-native English speakers, but the number of utterances is less than the Audio-Snips dataset. We omitted 34 speech data (31, 3, 1) that were not recognized by the ASR model from the training, validation, and test set, respectively. Consequently, we used 23,101 samples from the training set, 3084 samples from the development set, and 3791 samples from the test set. Each sample contained one sentence.

The Audio-Snips dataset is an audio-recorded version of the text-based intent classification dataset SNIPS [27]. The recorded voice files in the Audio-Snips datasets are made via an AI synthesizer, Amazon Polly. There are adults, children, and native speakers whose mother tongues are rooted in the United States and non-native speakers who are born in the USA but their parents are from non-English speaking countries. In other words, Audio-Snips contains a total of 16 speakers; 8 are synthesized with US English and the rest speak other varieties of English. These attributes of the datasets are able to lead the ASR system to generate various features of errors in the recognized text. The speech data for each speaker is composed of 13,084 training samples, 700 development samples, and 700 test samples, each of which contains one phrase. More information is available online: https://github.com/aws-samples/aws-lex-noisy-spoken-language-understanding (accessed on 29 September 2020).

### 3.2. Dataset Statistics

**Figure 2:**
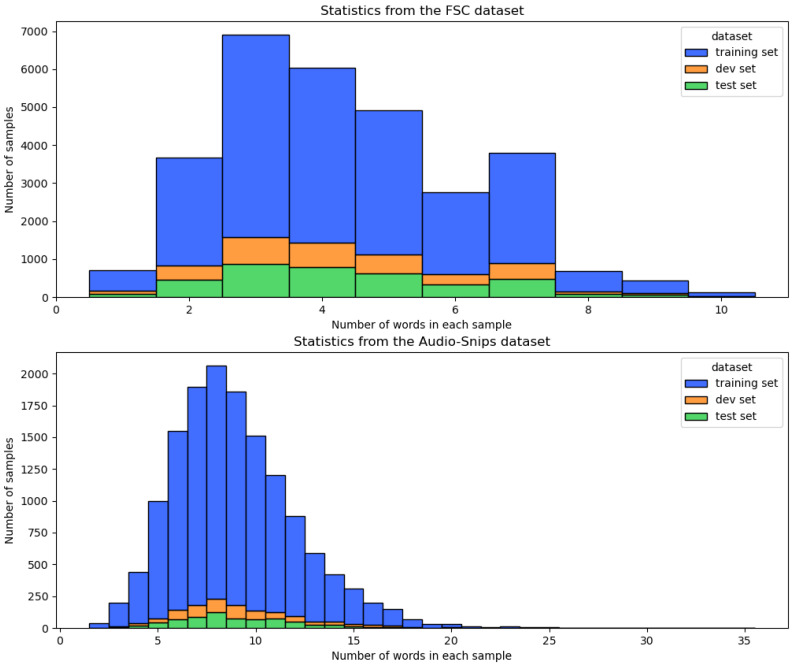
Distribution of words per sample in the FSC and the Audio-Snips datasets.

Figure 2 shows the statistics of the two datasets. Overall samples in the FSC dataset have a range of one to ten words. In particular, samples that contain two to four words are the most common. Since the FSC dataset is aimed at operating smart home devices, such as AI speaker appliances, the samples have shown that they are used in limited locations in a house, such as a kitchen, a living room, and a bathroom. For the same reason, the average utterance length of the FSC dataset is relatively short in comparison to the Audio-Snips dataset.

Here, since the Audio-Snips dataset was synthesized into the same text, we analyzed only one of them. As shown in the bottom part of Figure 2, most of the training, development, and test samples of the Audio-Snips dataset are three times longer than the FSC dataset. As with the FSC dataset, the Audio-Snips dataset contains samples that are mainly spoken to start and awake smart home devices. However, the respective training, development, and test files have more pronouns compared to the FSC dataset. In particular, the commands in the Audio-Snips dataset have names of singers, restaurants, and locations, respectively. Therefore, we conjecture that the ASR errors will be bound to occur more frequently in the Audio-Snips dataset than the FSC dataset.

### 3.3. Analyses on ASR Errors of Two Datasets

To observe the recognition errors in the spoken dialogue system, we report the ASR errors of the two datasets recognized by the Wav2vec 2.0 model. As shown in Table 1, in Audio-Snips, languages that Amazon-Polly provide are diverse, so the recognition results of native English speakers and non-native speakers were obtained separately. In the FSC dataset, however, since the types of speakers were not separated clearly, we denoted them as ‘All’.

In Table 1, it is observed that the WER of the US English speakers is 6.12% and 5.73% lower than that of non-US English speakers in the Audio-Snips training and test sets, respectively. In addition, we find that the ASR errors of male speakers are higher than that of female speakers, and this trend also applies to children. In particular, the lowest WER is from Kimberly who speaks US English, while the highest WER belongs to Brian whose mother tongue is British English. The higher WER of the recognized text indicates that the ASR system is still vulnerable to variation in the style of speech.

On the other hand, compared to the Audio-Snips dataset, the FSC dataset shows much lower WER and CER. However, in the cases where the commands are shorter, the ASR error rates clearly indicate the shortcomings of conventional ASR systems.

Table 2 demonstrates examples of the labeled text, corresponding recognized text, and the intent class, respectively. The Audio-Snips example sentences of Table 2 are from the training files of Nicole whose WER was 43.99% on the training set. The five examples of two datasets demonstrate that ASR errors mainly occur on verbs and nouns. The most serious problem of the Audio-Snips dataset is the ASR errors in the pronouns that indicate the intents of the whole sentence. In addition, the words that refer to the names of locations show notable errors. These errors on those locations lead to critical misunderstandings for the agents who have to respond to its owner’s request. For example, because of errors in Table 2, such as By blon (Bedroom), Internal (Kitchen), and Vilum (Volume), the location information cannot be understood correctly. In addition, the errors in verbs such as Bigly (Decrease) and Ese (Use) that indicate the main intent cause the NLP model to misunderstand the meaning of the location words.

These errors in the recognized text frequently lead the AI agent to repeat the same dialogue, which finally causes user dissatisfaction. When the environmental noise is mixed with the users’ voices, the ASR errors become more critical than the errors provided in Table 2. Through this, it seems that unless the language models are trained with additional information of frequent ASR errors, impeccable understanding of the users’ intents is still stuck at the limited level in the spoken dialogue condition.

### 3.4. Correlation between ASR Errors and Intent Classification

Here, we explore the relationships between ASR errors and intent classification performance in dialogue-oriented conditions. We train the intent detection model with labeled text, then evaluate with both test sets of labeled and recognized text. To this end, we fine-tune a language representation for intent classification using the pre-trained language model [15].

Table 3 reports the correlations between ASR errors from the current state-of-the-art recognizer and intent classification performance. As we have hypothesized, the higher the WER, the lower the classification performance of the model. In Table 3, the highest WER is 52.94%, and the corresponding Brian’s intent classification performance is 75.86%. In addition, the models trained on the dataset of Joanna, Kendra, Kimberly, and Salli whose WER scores are among the lowest in Audio-Snips achieved relatively high performance. Consequently, as the ASR errors increase in spoken dialogue conditions, the performance of the intent detection model significantly decreases.

### 3.5. Need for Language Representation Using Recognized Text

So far, we found that the higher the WER, the lower the intent classification accuracy. Furthermore, we showed that the problems of misunderstanding the users’ intents in the spoken dialogue systems frequently occurred due to the errors of the recognized text. As shown in Table 3, the intent accuracy of the test with labeled text is close to perfect.

To solve these problems, it is required to construct language models that are not only robust to errors in recognized text, but also work well in the labeled text corresponding to the original text dataset. To this end, we propose the method that jointly fine-tunes two language models with both labeled text and recognized text.

## 4. Methodology

In Section 3, we reported explicit results and analyzed each fine-tuned BERT model with both labeled and recognized text, and the corresponding intent classification result. In this section, we propose our model to elevate the limited ability of the spoken dialogue system.

### 4.1. Reasons for Utilizing the Pre-Trained Language Model

Models that are employed for our proposed method are pre-trained language models whose main architectures are composed of the encoder of the transformer. There are some reasons for employing pre-trained language models. Scratch training of a language model not only consumes a lot of computational resources, but it also requires an enormous amount of training datasets. Since our proposed method is aimed at improving the performance of language models using small amounts of data in pairs of labeled and recognized text, pre-trained models are an essential part of the downstream training phase.

In addition, the advantage of using the upstream model as a downstream task is that it absorbs additional information easier than other language models trained from scratch. The language model that has been pre-trained for powerful representation learning with a large amount of data can adapt to the recognized texts where errors are prevalent, and the fine-tuned model may be able to keep a good performance for any dialogue task.

### 4.2. Training Phase

In this section, we propose a method to improve the performance of the intent classification model in spoken dialogue conditions. Hereafter, given the user’s voice $v=[v_{1},...,v_{T}]$, the labeled text sentence corresponding to *v* is $s=[s_{1},...,s_{N}]$, recognized text sentence of *v* obtained by the ASR system is $\hat{s}=\left[{\hat{s}}_{1},...,{\hat{s}}_{M}\right]$, (*s*, $\hat{s}$) ∈ (*S*, $\hat{S}$), and the intent label of both *S* and $\hat{S}$ is *Y*.

Training the language model with the MLM approach, as is done in BERT is a self-supervised learning method to extract powerful representations, making it easy to leverage them in several downstream tasks. In this paper, we only consider the MLM, not the next sentence prediction task used in BERT. The objective function of the MLM scheme in pre-training BERT is

(1) $$L\left(\theta ;S\right)=\frac{1}{\left|S\right|}\sum_{s\in S}\log P\left(s^{s}|s^{m};\theta \right),$$

where $s^{s}$ is the selected tokens of each sentence *s* in the dataset, $s^{m}$ is the masked or replaced token of *s* selected by MLM policy, and θ is the model parameters. Equation (1) can be represented as

(2) $$M=\frac{1}{\left|S\right|}\sum_{s\in S}\log\prod_{n=1}^{N}P\left(s_{n}^{s}|s_{<n}^{s},s^{m};\theta \right).$$

The goal of the downstream task of classifying the intents is to find the optimal mapping function as follows:

(3) f:S→Y.

Suppose the “well-trained” of the intent classification model using M in Equations (2) and (3) is obtained as

(4) f(M)=argmaxP(Y|S).

Here, if the recognition errors are included in *S*, the model f(M) cannot perform well owing to

(5) $$P\left(Y|\hat{S}\right)\leq P\left(Y|S\right).$$

$\hat{S}$ can have insertion, deletion, substitution and transposition errors compared to *S*, so the model f(M) in Equation (4) cannot know the perceived ASR errors in the spoken dialogue conditions.

**Figure 3:**
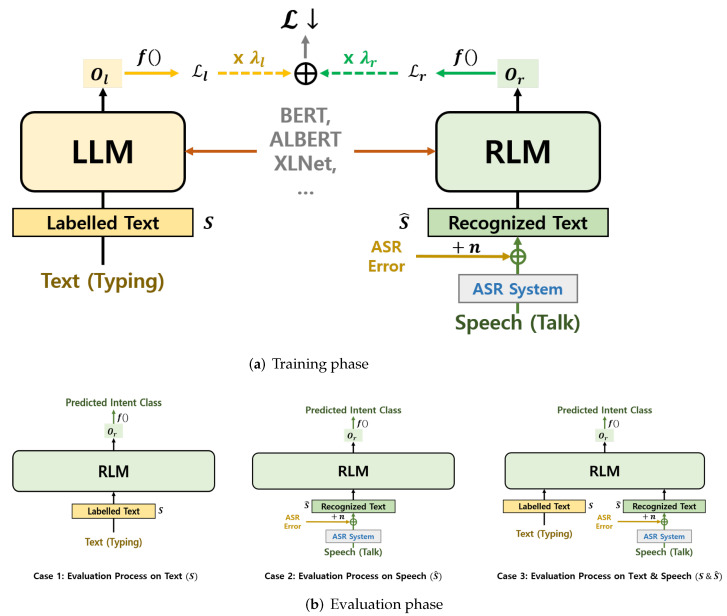
Model architecture of our proposed model. The training phase of our model shown in (**a**) uses the labeled text and recognized text jointly to train the users’ intents. In the evaluation process, we only use the RLM to find optimal users’ needs as illustrated in (**b**). As shown in (**b**), the input of the proposed model can be any combination of text and voice (recognized text).

To cope with the confusing problems of intent classification in the dialogue system, our goal was to make the intent classification models f(M) achieve robust performance for $\hat{S}$ without losing information on *S*. To this end, we suggest a method that jointly fine-tunes two pre-trained language models. As shown in Figure 3a, the proposed model is composed of two main components, which are LLM (labeled language model) and RLM (recognized language model). The proposed method optimizes with *S* and $\hat{S}$ to find $f\left(M\right):S+\hat{S}\to Y$.

During training, both labeled text (*S*) and recognized text ($\hat{S}$) are given, the LLM and the RLM of the proposed model are gradually and jointly trained to classify the intents. In other words, the main purpose of fine-tuning two pre-trained models is to set the RLM as a ***handler*** for $\hat{S}$. The purpose of the handler is formulated as

(6) $$P\left(Y|\hat{S}\right)\approx P\left(Y|S\right).$$

Given the input sentences from $\hat{S}$, the handler works to guess the misspelled words that are defined in the pre-trained tokenizer. Common errors in spelling are largely categorized into four parts; insertion, deletion, substitution, and transposition of characters in a word. Specifically, insertion in misspelling means unexpected characters are added in a word, deletion in the spelling errors is the omission of certain characters in a word, and substitution means a character in a word is replaced with another character, which causes misspelling errors. Lastly, transposition in misspelling errors means that the order of words is changed.

To set up a robust language model that is sufficient to guess the wrong words in the spoken dialogue system, two language models are fine-tuned with both *S* and $\hat{S}$. In particular, the backpropagation process is important because each model can become aware of the text information from *S* and $\hat{S}$. To this end, the loss from the LLM and RLM is essential to update the models to handle the two mismatched types of texts. When the losses are computed from two models, the final loss is summed with weight coefficients denoted as λ, which has a certain ratio for the respective LLM and RLM. The objective function of the proposed model is formulated as

(7) $$L=L_{l}\lambda_{l}+L_{r}\lambda_{r},$$

where both $L_{l}$ and $L_{r}$ are as

(8) $$L_{l}=-\sum_{i=1}^{N}t_{i}\log\left(f{\left(o_{l}\right)}_{i}\right)$$

(9) $$L_{r}=-\sum_{j=1}^{M}t_{j}\log\left(f{\left(o_{r}\right)}_{j}\right),$$

where $o_{l}$ and $o_{r}$ are the final outputs of the LLM and RLM before the softmax function. The outputs of softmax $f{\left(o_{l}\right)}_{i}$ and $f{\left(o_{r}\right)}_{j}$ are as below:

(10) $$f{\left(o_{l}\right)}_{i}=\frac{e^{o_{l_{i}}}}{\sum_{K}e^{o_{l_{k}}}}$$

(11) $$f{\left(o_{r}\right)}_{j}=\frac{e^{o_{r_{j}}}}{\sum_{K}e^{o_{r_{k}}}}.$$

Then, the calculated loss is passed to both of the models so that they simultaneously learn *S* and $\hat{S}$. The two models are gradually trained to notice the unseen information, which was not provided during the forwarding process. $\lambda_{l}$ and $\lambda_{r}$ in Equation (7) are the hyper-parameters of adjusting the ratio of each logit and they are designed as follows:

(12) $$\lambda_{l}+\lambda_{r}=1.0.$$

The effects of different applications of the λ ratio on the proposed method are demonstrated in Section 5.5.

To sum up, the proposed approach can optimally map both speech and text corresponding to the users’ intents in practical scenarios. Furthermore, while most of the earlier ASR-SLU-based studies required large computational costs and complex methods to understand users’ intents with end-to-end architecture, our proposed method circumvents substantial computation costs and complicated methods. Our model is able to quickly and accurately perform the downstream tasks using the pre-trained model, which is learned with the large-scale dataset.

### 4.3. Evaluation Phase

Our evaluation process is shown in Figure 3b. Since the LLM and RLM are jointly updated using the objective function, the RLM is gradually exposed to these values and can learn linguistic information. Due to the advantages of this downstream task, the RLM shows strong intent classification performance on $\hat{S}$ without deterioration on *S*. Furthermore, users can input their intents to our suggested model by using speech (recognized text), text, or together. In practice, the proposed method will show results that overcome the shortcomings and outperform the existing solutions in Section 5.

## 5. Experiment and Result Analysis

### 5.1. Experiment Setting

We used the FSC dataset and Audio-Snips dataset, which are described in Section 3. For ASR, we directly applied the pre-trained Wav2vec 2.0 (available online: https://huggingface.co/docs/transformers/model_doc/wav2vec2, accessed on 9 June 2021) from huggingface [28] without additional training. In addition, the BERT used in all of our experiments was fine-tuned until 20 epochs with a learning rate of 1e-4, using the batch size of 64 with the Adam optimizer [29]. To fine-tune BERT, we set $\lambda_{l}=0.55$ and $\lambda_{r}=0.45$ in the FSC dataset experiments, and, $\lambda_{l}=0.85$ and $\lambda_{r}=0.15$ in the Audio-Snips dataset.

We also utilized the following models that were released on the huggingface [28]: ALBERT [16], XLNet [17], ELECTRA [18], RoBERTa [19].

### 5.2. Results

Table 4 illustrates the experimental results of applying our method to Audio-Snips and FSC datasets. Further, four evaluation results of the conventional single model approach are provided (two of them were obtained by the model trained with *S* and the others from the model trained with $\hat{S}$). Consequently, we show the performance evaluation using three methods (trained with *S*, $\hat{S}$, and $\left(S+\hat{S}\right)$ (Ours)) to compare intent classification on five pre-trained language models. The symbol “→” in Table 4 means an operation that is trained with the preceding training set and evaluated with the following test set. For example, “$S\to \hat{S}$” refers to the process of training with *S* and evaluating with $\hat{S}$. In the case of our proposed model, the operations are denoted as $\left(S+\hat{S}\right)\overset{\mathrm{Ours}}{\to }S$ and $\left(S+\hat{S}\right)\overset{\mathrm{Ours}}{\to }\hat{S}$.

In the Audio-Snips dataset, there are five models that achieved an average of 98.11% and 88.24% accuracy on both S→S and $S\to \hat{S}$. In the FSC dataset results, the average accuracy of S→S and $S\to \hat{S}$ are 100% and 87.55%. In each dataset, fine-tuned models trained on *S* show about 10% accuracy difference in the evaluations of *S* and $\hat{S}$.

According to these results, we strongly conjecture that the performance of $S\to \hat{S}$ would be significantly decreased in practical spoken dialogue scenarios where more noise and extreme conditions exist. In other words, these results indirectly explain why the text-based intent classification system cannot be used in the practical spoken dialogue system and the reasons that recent studies focused on the ASR-SLU-based approaches. Therefore, it is required to improve the spoken language model performance, not only to satisfy users’ needs, but also to overcome the limitations of conventional SLU approaches.

On the other hand, in the Audio-Snips dataset, the average accuracy of $\hat{S}\to S$ and $\hat{S}\to \hat{S}$ achieved 97.80% and 96.95%, respectively. Compared to both average accuracy of S→S and that of $S\to \hat{S}$, the accuracy of $\hat{S}\to S$ is 0.31% lower and that of $\hat{S}\to \hat{S}$ is 8.71% higher. In the FSC dataset, the average accuracy of $\hat{S}\to S$ and $\hat{S}\to \hat{S}$ are 98.13%, 95.49%. The results of the FSC dataset show the same trend as with the Audio-Snips. Although the average intent classification performance of $\hat{S}$ is significantly enhanced in $\hat{S}\to \hat{S}$ compared to the models trained with *S*, there is a problem that the average accuracy of $\hat{S}\to S$ is lower than that of $S\to \hat{S}$. Moreover, it is still a concern that $\hat{S}\to \hat{S}$ results are not robust. Compared to the average performance of S→S (100%) in the FSC dataset, the average $\hat{S}\to \hat{S}$ performance is 4.51% lower (95.49%), which can be a critical problem in special intent understanding. Therefore, it is important to incorporate the linguistic information of *S* in training of $\hat{S}$.

In the results of our proposed method, we achieve the average accuracies from both *S* and $\hat{S}$ as 98.94% and 98.26% in the Audio-Snips dataset. These results show that the proposed method yields better performance than the matched condition $S\to \hat{S}$. In the results of the FSC dataset, our method gets the highest average performances, which are 100% and 98.64%. In particular, $\left(S+\hat{S}\right)\overset{\mathrm{Ours}}{\to }\hat{S}$ outperforms $S\to \hat{S}$ by 11.09%. In view of practical spoken dialogue systems, the proposed method demonstrates that the difference between the performance of *S* and $\hat{S}$ has narrowed significantly compared to the other results. Through the results on the two datasets, it is clear that the proposed method can generalize to the various conditions in spoken dialogue systems. Our proposed method showed the performance on the recognized text that outperforms the other approaches, while the performance on labeled text did not deteriorate, but rather improved. Owing to the linguistic information from RLM and the attributes of the pre-trained language models, our proposed method demonstrated robustness and efficiency in both *S* and $\hat{S}$ of Audio-Snips and FSC dataset.

In addition, unlike the non-explainable end-to-end process of the ASR-SLU models, the proposed model is able to clarify the error analysis in a dialogue-oriented system. Moreover, it enables the user interfaces to use both speech and text. We expect the proposed method to show strong performance even in practical scenarios where unexpected noise and various speakers exist.

### 5.3. Comparisons to Other Studies

To validate the effectiveness of our proposed method, we compare it to other approaches using the Audio-Snips dataset.

In the experiments based on the Audio-Snips dataset, three earlier studies were selected. Table 5 demonstrates the results of the proposed method and the conventional methods. Note that both Huang et al. [9] and Cao et al. [10] used the recognized text, and Lai et al. [13] set the input as speech. For the case using recognized text from the ASR system, the proposed method outperforms other methods. It shows the best performance when speech data itself are used as input, but this end-to-end method requires a lot of computation. The proposed method shows comparable performance to Lai’s approach [13] through a simple spoken language representation. As shown in Table 5, the average accuracy of our proposed model achieved the highest performance in the Audio-Snips dataset.

### 5.4. Analysis

In this section, we analyze the effects of the proposed models, focusing on the performance of recognized text. To this end, we compare using the prediction results and confusion matrices of $S\to \hat{S}$ and $\left(S+\hat{S}\right)\overset{\mathrm{Ours}}{\to }\hat{S}$, respectively. In the Audio-Snips dataset, we select the two speakers with the worst WER. In the FSC dataset, we visualize all results.

Table 6 shows the test samples of Brian, Russell, and the full FSC dataset for analysis. In both Brain and Russell, $\hat{S}$ is significantly different from *S*. In particular, as we observed in Section 3, the recognized text of verb and noun parts have many ASR errors. As shown in Table 6, $\hat{S}$ is not predicted well from the model trained with *S*. These lead to weak SLU models and cause misclassifying of users’ intents in practical spoken dialogue systems.

On the other hand, our results ($\left(S+\hat{S}\right)\overset{\mathrm{Ours}}{\to }\hat{S}$) in Table 6 demonstrated better results. Although many ASR errors exist in $\hat{S}$, our proposed method can find the users’ intentions well owing to the RLM. However, we conjecture that the word “see” in Russell’s fourth sample makes the model confused; this made the proposed model misclassify “AddToPlaylist” as “SearchScreeningEvent”.

**Figure 4:**
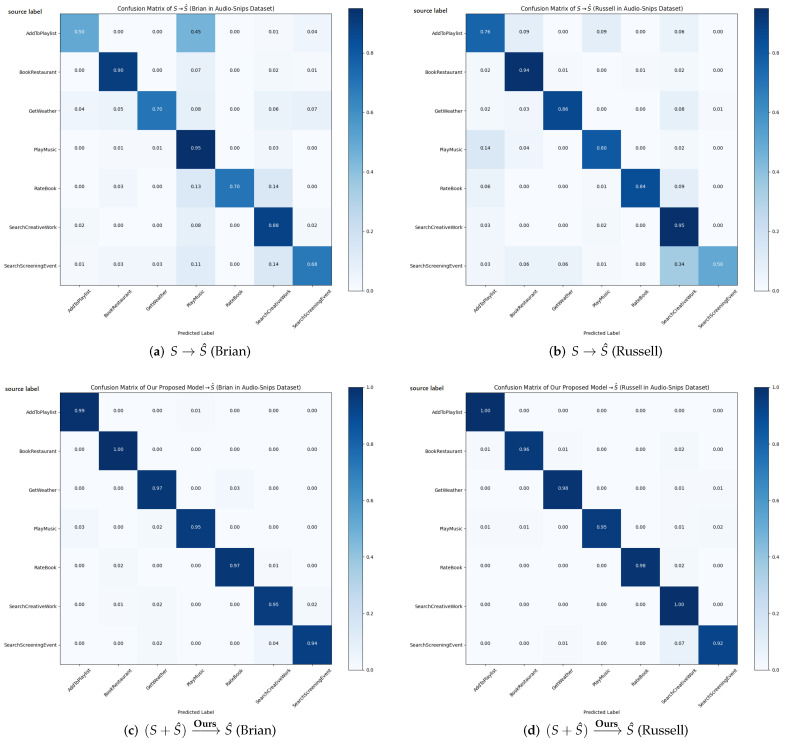
Confusion matrices of intent accuracy from Brian and Russell in the Audio-Snips dataset. (**a**,**b**) are $S\to \hat{S}$ of Brian and Russell and (**c**,**d**) are their $\left(S+\hat{S}\right)\overset{\mathrm{Ours}}{\to }\hat{S}$, respectively.

Figure 4 provides the confusion matrices corresponding to both the results of Brian and Russell in Table 6 for a more detailed analysis. As shown in Figure 4a,b, even though there are only seven intent classes, more than half of them do not exceed 90% accuracy. Specifically, in Figure 4a,b, the accuracy of label “AddToPlaylist” are 0.50 and 0.76, and that of “SearchScreeningEvent” are 0.68 and 0.50. This is due to the recognition errors and it means that it is difficult to accurately classify the users’ intents in the noisy environment using models trained only with labeled text.

On the other hand, the results of $\left(S+\hat{S}\right)\overset{\mathrm{Ours}}{\to }\hat{S}$ in both Figure 4c,d show that all of the intent classes in each speaker achieved more than 92% accuracy. While the accuracy of “AddToPlaylist” in Figure 4a and that of “SearchScreeningEvent” in Figure 4b have 0.50, our results in Figure 4c,d are 0.99 and 0.92, respectively. In addition, while $S\to \hat{S}$ operation for Brian and Russell achieved 75.86% and 80.75% accuracy, the proposed method achieved 96.71% and 97% accuracy, which outperformed by 20.85% and 16.29%, respectively. Consequently, these results validate that the proposed method is effective for classifying intents in the spoken dialogue system containing severe ASR errors.

### 5.5. Ablation Studies

In this section, we provide the results of the ablation studies to explicitly investigate the performance of our proposed model under various configurations. The first ablation study is focused on different hyperparameter values, and the second one utilizes different methods in the fine-tuning procedures, such as masking, token-level mix-up [30].

For better reproducibility, we report specific values of hyperparameters. Table 7 shows the performance comparisons for two different λ values. In the case of the Audio-Snips dataset, the optimal values for $\lambda_{l}$ and $\lambda_{r}$ are 0.85 and 0.15 for BERT, ELECTRA, and RoBERTa, while for ALBERT and XLNet, they are 0.55 and 0.45. For all models in the FSC dataset, the optimal values for $\lambda_{l}$ and $\lambda_{r}$ are 0.55 and 0.45.

As described in Table 7, all accuracies are similar regardless of the hyperparameters. Since the proposed method is evaluated using RLM, we found that the best accuracy is obtained when the value of $\lambda_{l}$ is higher than that of $\lambda_{r}$ in order to share more attributes of the LLM with the RLM.

Table 8 shows the results of using various augmentation methods to make a robust SLU model. First, we applied the masking method in fine-tuning. To this end, in the training phase, 10%, 20%, and 30% of the total words were replaced with “<MASK>” token, while no masking was used in the evaluation phase. Second, we used the mix-up [30] approach at the token level and applied it within the range allowed by the vocabulary size of the pre-trained tokenizer. Both mentioned methods were applied to *S* and evaluated on $\hat{S}$. In addition, the experiment was conducted with a single BERT model, and the two methods are denoted as $S_{Mask_{\left[10,20,30\right]}}\to \hat{S}$ and $S_{Mix}\to \hat{S}$, respectively.

As shown in Table 8, the performance of the models fine-tuned with the different ratio of masking approaches ($S_{Mask_{10}}\to \hat{S}$, $S_{Mask_{20}}\to \hat{S}$, $S_{Mask_{30}}\to \hat{S}$) are higher than that of $S\to \hat{S}$, but lower than ours ($\left(S+\hat{S}\right)\overset{\mathrm{Ours}}{\to }\hat{S}$). Furthermore, training with the mix-up approach ($S_{Mix}\to \hat{S}$) yielded the worst performance. According to this analysis, we find that neither the mix-up approach nor the masking method is effective for the improvement of performance. Consequently, the results in Table 8 show that our proposed model outperforms the other augmentation-based methods.

## 6. Conclusions

Thus far, we have observed the limited ability of existing spoken dialogue systems in practical scenarios. In this paper, we suggested a method to address a significant problem involving the spoken dialogue system due to recognized errors that are generated by the ASR system in practical scenarios. Our novel and simple method fine-tunes spoken language models jointly using recognized text and labeled text. The experimental results show that our proposed RLM maintains high accuracy on labeled text while improving the performance of intent classification on recognized text. Our proposed method can successfully understand users’ intentions for practical dialogue-oriented systems and allow users to transfer their intents to the spoken dialogue system with only speech or text alone, if necessary.

## Figures and Tables

**Table 1:** Observations of both CER and WER of Audio-Snips and FSC dataset, respectively. The lower the CER and WER means the better performance of the ASR. Note that both CER and WER are measured without punctuation (e.g., !, ?, ., ‘).

Dataset	Speaker	Language	Gender	Training Set		Test Set	
				CER (%)	WER (%)	CER (%)	WER (%)
Audio-Snips	Aditi	Hindi	Female	18.04	41.71	17.60	40.92
	Amy	English (British)	Female	15.25	35.55	14.66	34.72
	Brain	English (British)	Male	25.22	52.94	24.44	50.72
	Emma	English (British)	Female	14.89	34.84	14.16	33.76
	Geraint	English (Welsh)	Male	21.81	48.37	21.04	45.93
	Ivy	English (US)	Female (child)	14.85	35.30	14.48	34.95
	Joanna	English (US)	Female	14.15	33.06	13.41	32.03
	Joey	English (US)	Male	19.83	43.39	19.16	42.28
	Justin	English (US)	Male (child)	19.44	44.06	18.38	42.17
	Kendra	English (US)	Female	13.78	33.06	13.11	32.18
	Kimberly	English (US)	Female	13.49	32.89	12.85	31.90
	Matthew	English (US)	Male	20.96	45.62	20.12	43.87
	Nicole	English (Australian)	Female	19.69	43.99	19.00	42.02
	Raveena	English (Indian)	Female	18.24	41.69	17.93	41.42
	Russell	English (Australian)	Male	24.00	51.17	22.57	48.40
	Salli	English (US)	Female	14.19	33.87	13.54	32.68
	En-US (average)			16.34	37.66	15.63	36.51
	Non En-US (average)			19.64	43.78	18.93	42.24
	All (average)			17.99	40.72	17.28	39.37
FSC	All			12.75	27.20	5.58	14.26

**Table 2:** Examples of the labeled text, the recognized text, and the intent label of the Audio-Snips and FSC dataset, respectively.

Dataset	#	Labeled Text	Recognized Text	Intent
Audio-Snips	1	Rate this album a 1	Great thi salbumawan	RateBook
	2	Can you tell me the weather forecast for six am in grenada	Can ye tell me the wether folcasfa seek samin grenado	GetWeather
	3	Book a spot for oneat the wolseley at elevenses	Bokust spot for one at the wosli atalevanses	BookRestaurant
	4	Book a table for doris and i in new tulsa	Bucetabu fidarus and iin neutalsa	BookRestaurant
	5	Use groove shark to play the today and tomorrow album	Ese grieve shock to play the to day and to morrow album	PlayMusic
FSC	1	Decrease the heating in the bathroom	Bigly then heaping in the budroom	Decrease-heat-washroom
	2	Kitchen heat up	Internal heed of	Increase-heat-kitchen
	3	Volume up	Vilum a	Increase-volume-none
	4	Bedroom heat down	By blon heap don	Decrease-heat-bedroom
	5	Switch off the lights	Of the lats	Deactivate-lights-none

**Table 3:** Observations of correlations between recognized errors (WER) and intent classification performance. Each intent accuracy (Acc) in table was obtained by the BERT model that was trained with labeled training text and evaluated with the test set of each labeled and recognized text, respectively.

Dataset	Speaker	WER (%)		Intent Acc of the BERT Trained with Labeled Text (%)	
		Training Set	Test Set	Test with Labeled Set	Test with Recognized Set
Audio-Snips	Aditi	41.71	40.92	98.43	84.71
	Amy	35.55	34.72		90.86
	Brian	52.94	50.72		**75.86**
	Emma	34.84	33.76		90.43
	Geraint	48.37	45.93		82.29
	Ivy	35.30	34.95		93.85
	Joanna	33.06	32.03		92.29
	Joey	43.39	42.28		87.42
	Justin	44.06	42.17		85.29
	Kendra	33.06	32.18		93.86
	Kimberly	32.89	31.90		94.57
	Matthew	45.62	43.87		84.57
	Nicole	43.99	42.02		85.43
	Raveena	41.69	41.42		83.57
	Russell	51.17	48.40		80.71
	Salli	33.87	32.68		**94.86**
	En-US	37.66	36.51		90.84
	Non En-US	43.78	42.24		84.23
	Average	40.72	39.37		87.54
FSC	All	27.20	14.26	100	89.63

**Table 4:** Experiment results on both the Audio-Snips dataset and FSC dataset.

Intent Classification Acc (%)							
**Dataset**	**Used** **Model**	S→S	$S\to \hat{S}$	$\hat{S}\to S$	$\hat{S}\to \hat{S}$	$\left(S+\hat{S}\right)\overset{\mathrm{Ours}}{\to }S$	$\left(S+\hat{S}\right)\overset{\mathrm{Ours}}{\to }\hat{S}$
Audio-Snips	BERT	98.29	89.77	98.29	96.96	99.43	98.62
	ALBERT	97.86	87.29	97.71	96.84	98.29	97.97
	XLNet	98.14	89.41	97.57	97.31	99.29	98.43
	ELECTRA	98.57	89.58	98.14	97.21	99.14	98.52
	RoBERTa	97.71	85.15	97.31	96.44	98.57	97.76
	Average	98.11	88.24	97.80	96.95	98.94	98.26
FSC	BERT	100	88.81	98.50	96.23	100	98.63
	ALBERT	100	85.65	98.39	95.86	100	98.68
	XLNet	100	88.16	99.14	96.97	100	98.66
	ELECTRA	100	85.73	96.94	93.38	100	98.63
	RoBERTa	100	89.40	97.68	94.99	100	98.60
	Average	100	87.55	98.13	95.49	100	98.64

**Table 5:** Intent classification performances of comparative models and ours in the Audio-Snips dataset experiments.

Model	Intent Acc (%)	Input
Huang et al. [9]	89.55	Recognized text
Cao et al. [10]	98.60	Recognized text
Lai et al. [13]	98.65	Speech
**Ours (BERT)**		
$\left(S+\hat{S}\right)\overset{\mathrm{Ours}}{\to }S$	99.43	Labeled text
$\left(S+\hat{S}\right)\overset{\mathrm{Ours}}{\to }\hat{S}$	98.62	Recognized text
Average	99.03	Labeled & Recognized text

**Table 6:** Detailed results analysis of Brian, Russell, and the FSC dataset.

Dataset	Speaker	#	Labeled Text	Recognized Text	Label	Prediction	
						$S\to \hat{S}$	$\left(S+\hat{S}\right)\overset{\mathrm{Ours}}{\to }\hat{S}$
Audio-Snips	Brian	1	Book a reservation at tavern for noodle	Bork te resurvation of tavernfemoodl	BookRestaurant	PlayMusic	BookRestaurant
		2	Add brazilian flag anthem to top 100 alternative tracks on spotify	Ad brazilian flagamthum to top 100 alternative tracks on spotify	AddToPlaylist	PlayMusic	AddToPlaylist
		3	Tell me the weather forecast for gibsland	Tell me the worther forecast for gibsland	GetWeather	SearchCreativeWork	GetWeather
		4	Rate this current album 0 starts	Break this caran talbon’s ero stars	RateBook	PlayMusic	RateBook
		5	Add abacab to beryl’s party on fridays playlist	A dabicabto party on friday’s playlist	AddToPlaylist	PlayMusic	AddToPlaylist
	Russell	1	Let’s hear good mohammad mamle on vimeo	Lets heer good mahammed mammelon venier	PlayMusic	AddToPlaylist	PlayMusic
		2	What is the weather forecast for burundi	Orders the wore forme cost or branby	GetWeather	BookRestaurant	GetWeather
		3	Where can i find the movie schedules	Where can i find the murvy schedules	SearchScreeningEvent	SearchCreativeWork	SearchScreeningEvent
		4	Add farhad darya song in virales de siempre	Out far hou die your songs in the rowls to see em prey	AddToPlaylist	SearchScreeningEvent	SearchScreeningEvent
		5	Add we have a theme song to my house afterwork	Ad we have a theme song to my house off to wor	AddToPlaylist	SearchScreeningEvent	AddToPlaylist
FSC	All	1	Turn down the heat in the washroom	Turn down we heet in the wateroom	decrease-heat -washroom	decrease-volume -none	decrease-heat -washroom
		2	Heat down	Keep down	decrease-heat -none	decrease-volume -none	decrease-heat -none
		3	Volume up	Fall him up	increase-volume -none	deactivate-lights -none	increase-volume -none
		4	Washroom lights off	Washed him lights off	deactivate-lights -washroom	deactivate-lights -none	deactivate-lights -washroom
		5	I need to practice my korean switch the language	I need to practice my careean which the language	change language -Korean-none	change language -English-none	change language -Korean-none

**Table 7:** Performance comparison of intent classification according to the different values of hyperparameters in two datasets.

Dataset	λ		$\left(S+\hat{S}\right)\overset{\mathrm{Ours}}{\to }\hat{S}$ Performance (%)				
	$\lambda_{l}$	$\lambda_{r}$	BERT [15]	ALBERT [16]	XLNet [17]	ELECTRA [18]	RoBERTa [19]
Audio-Snips	0.85	0.15	98.62	97.72	98.10	98.52	97.76
	0.55	0.45	98.08	97.97	98.43	98.34	93.34
	0.50	0.50	98.07	97.77	98.13	98.17	96.63
	0.45	0.55	98.19	97.83	97.74	98.33	96.98
	0.15	0.85	98.29	97.75	97.72	98.14	96.38
FSC	0.85	0.15	98.60	98.44	98.66	98.55	98.55
	0.55	0.45	98.63	98.68	98.66	98.63	98.60
	0.50	0.50	98.43	98.44	98.58	98.52	98.52
	0.45	0.55	98.52	98.37	98.47	98.52	98.47
	0.15	0.85	98.49	98.18	98.47	98.39	98.52

**Table 8:** The results of different augmentation approaches used in the downstream task.

Method (BERT)	Acc (%)	
	Audio-Snips	FSC
$S\to \hat{S}$	89.77	88.81
$S_{Mask_{10}}\to \hat{S}$	93.78	93.06
$S_{Mask_{20}}\to \hat{S}$	94.28	92.51
$S_{Mask_{30}}\to \hat{S}$	93.81	93.01
$S_{Mix}\to \hat{S}$	77.92	89.61
$\left(S+\hat{S}\right)\overset{\mathrm{Ours}}{\to }\hat{S}$	98.26	98.63

## Data Availability

The Audio-Snips dataset is available at https://github.com/aws-samples/aws-lex-noisy-spoken-language-understanding (accessed on 29 September 2020) and the FSC dataset is available at [7].
